# The skin microbiome of elasmobranchs follows phylosymbiosis, but in teleost fishes, the microbiomes converge

**DOI:** 10.1186/s40168-020-00840-x

**Published:** 2020-06-13

**Authors:** Michael P. Doane, Megan M. Morris, Bhavya Papudeshi, Lauren Allen, Dnyanada Pande, John M. Haggerty, Shaili Johri, Abigail C. Turnlund, Meredith Peterson, Dovi Kacev, Andy Nosal, Deni Ramirez, Kevin Hovel, Julia Ledbetter, Amanda Alker, Jackeline Avalos, Kristi Baker, Shruti Bhide, Emma Billings, Steven Byrum, Molly Clemens, Amelia Juliette Demery, Lais Farias Oliveira Lima, Oscar Gomez, Omar Gutierrez, Selena Hinton, Donald Kieu, Angie Kim, Rebeca Loaiza, Alexander Martinez, Jordan McGhee, Kristine Nguyen, Sabrina Parlan, Amanda Pham, Rosalyn Price-Waldman, Robert A. Edwards, Elizabeth A. Dinsdale

**Affiliations:** 1 Sydney Institute of Marine Science, Mosman, NSW Australia; 2 Biology Department, San Diego State University, San Diego, CA USA; 3 Department Biology, Stanford University, Stanford, California, USA; 4 National Center for Genome Analysis Support, Indiana University, San Diego, Indiana USA; 5 Computer Sciences Department, San Diego State University, San Diego, CA USA; 6 Hopkins Marine Station, Stanford University, Pacific Grove, CA USA; 7 Australian Centre for Ecogenomics, The University of Queensland, St. Lucia, Queens, USA; 8 Scripps Institute of Oceanography, University of California-San Diego, La Jolla, California, USA; 9 Department of Environmental and Ocean Sciences, University of San Diego, San Diego, CA USA; 10 Whale Shark Mexico, ConCiencia Mexico AC, La Paz, BC USA; 11 Department of Biology, University of Florida, Gainesville, FL USA; 12 Department of Ecology and Evolutionary Biology, Princeton University, Princeton, NJ USA; 13 Viral Information Institute, San Diego State University, San Diego, CA USA

**Keywords:** Microbiome, Phylosymbiosis, Metagenomics, Elasmobranch skin, Teleost, Vertebrate fishes, Microbial community, Community ecology

## Abstract

**Background:**

The vertebrate clade diverged into Chondrichthyes (sharks, rays, and chimeras) and Osteichthyes fishes (bony fishes) approximately 420 mya, with each group accumulating vast anatomical and physiological differences, including skin properties. The skin of Chondrichthyes fishes is covered in dermal denticles, whereas Osteichthyes fishes are covered in scales and are mucous rich. The divergence time among these two fish groups is hypothesized to result in predictable variation among symbionts. Here, using shotgun metagenomics, we test if patterns of diversity in the skin surface microbiome across the two fish clades match predictions made by phylosymbiosis theory. We hypothesize (1) the skin microbiome will be host and clade-specific, (2) evolutionary difference in elasmobranch and teleost will correspond with a concomitant increase in host-microbiome dissimilarity, and (3) the skin structure of the two groups will affect the taxonomic and functional composition of the microbiomes.

**Results:**

We show that the taxonomic and functional composition of the microbiomes is host-specific. Teleost fish had lower average microbiome within clade similarity compared to among clade comparison, but their composition is not different among clade in a null based model. Elasmobranch’s average similarity within clade was not different than across clade and not different in a null based model of comparison. In the comparison of host distance with microbiome distance, we found that the taxonomic composition of the microbiome was related to host distance for the elasmobranchs, but not the teleost fishes. In comparison, the gene function composition was not related to the host-organism distance for elasmobranchs but was negatively correlated with host distance for teleost fishes.

**Conclusion:**

Our results show the patterns of phylosymbiosis are not consistent across both fish clades, with the elasmobranchs showing phylosymbiosis, while the teleost fish are not. The discrepancy may be linked to alternative processes underpinning microbiome assemblage, including possible historical host-microbiome evolution of the elasmobranchs and convergent evolution in the teleost which filter specific microbial groups. Our comparison of the microbiomes among fishes represents an investigation into the microbial relationships of the oldest divergence of extant vertebrate hosts and reveals that microbial relationships are not consistent across evolutionary timescales.

Video abstract

## Introduction

Phylogenetically diverse microorganisms (virus, bacteria, archaea, and micro-eukaryotes) colonize living surfaces. These organisms collectively form the microbiome, which is involved in processes such as host development [1], host nutrient provisioning [2], and disease resistance [3, 4]. The outer surfaces of marine organisms are exposed to millions of microbial cells. However, the skin microbiome is distinct from the surrounding water column microbes [5–7]. Therefore, the skin surface is selecting and regulating the microbiome. During homeostasis, the microbiome and host interact as a unit termed the holobiont and together maximize the ecological success of the host organism [8]. Holobionts are observed across diverse host organisms, ranging from invertebrates [8] to vertebrates [9]. The intimate relationships between many host-microbiomes have led to an extension of the holobiont concept to include an evolutionary perspective called phylosymbiosis. The phylosymbiosis concept suggests that hosts and microbiomes are linked eco-evolutionarily, such that the microbiome composition will recapitulate the host’s evolutionary trajectory [10]. Therefore, hosts that are more phylogenetically related will have microbial communities that are more closely related, whereas hosts with greater phylogenetic divergence will have microbiomes with dissimilar compositions. This evolutionary view assumes reciprocal benefits for the function of the holobiont in homeostasis. Gut-derived microbial communities of apes [11], ants [12], and bats [9] exhibit patterns that are consistent with those predicted by phylosymbiosis. The presence of phylosymbiotic patterns is hypothesized to be the result of vertical inheritance, defined as intra-species microbial transmission, including but not exclusive to paternal transfer [13]. Alternative processes can lead to microbial patterns where more similar host species share more similar microbiomes than distantly related species [14]. Some argue that patterns of phylosymbiosis arise purely through processes of environmental filter, as hosts that share a recent evolutionary ancestor are more likely to harbor phenotypes, whether physiological, behavioral, or ecological, which select similar microbes from their environment [15]. Many terrestrial host species have behavioral characteristic which bring individuals of the same species in close contact, yet patterns consistent with phylosymbiosis are present [16, 17].

Phylosymbiosis is reported more often from studies investigating internal compartments of the organism, such as the gut of mammals and the plant root endosphere, than from external surfaces, such as leaf and skin surfaces [18]. External surfaces across a wide range of species display selective processes, such that the surface microbiomes are more similar among individuals of the same species from the same location [6, 7, 19]. Tests for the relationship between epidermal microbiome similarity and the host’s evolutionary history are limited. Phylosymbiosis occurred in the epidermal microbiomes of mammals [17], and there was weak support in the microbiomes of coral reef fishes [20]. In contrast to phylosymbiosis, the skin microbiomes of several amphibian species reflects the host’s ecology rather than host phylogeny [21]. The skin microbiome of the amphibians differed from the surrounding environment. However, amphibians from the same habitat had similar microbiomes regardless of their phylogenetic relationship, suggesting interactions of both environment and host selection processes. Diet also contributes to the skin microbiome structure as dietary factors influence the surface condition, such as the presence of oily secretions [22]. The skin microbiome of coral reef fishes showed weak evidence of phylosymbiosis but also correlated with fish diet. Understanding the factors which result in skin microbiome patterns is an important step to understanding ecological and evolutionary succession of the host microbiome in light of changing environmental conditions, which are suspected to influence microbial pools [23] and, thus microbes available to be recruited to the surface microbiome. For example, Alphaproteobacteria, a class of bacteria associated with marine teleost and elasmobranchs [6, 24], is sensitive to changes in temperature and *p*CO2 [19, 25].

The most abundant group of marine vertebrates is the fishes, including Chondrichthyes (cartilaginous) and Osteichthyes (bony or ray-finned) fishes. These fishes diverged approximately 420 mya [26], and the two clades have accumulated vast anatomical and physiological differences [27–29], particularly in the skin organ. For instance, the white shark genome analysis confirmed the presence of genes responsible for fast wound healing of the skin structure. A notable difference among the two clades is the presence of dermal denticles on elasmobranch skin, which are skin protrusions composed of material similar to teeth [30]. Ray-finned fishes (from here on teleost), however, have scales derived from keratinized epithelial tissue and a layer of mucus [28]. The different skin structures of these two distant fish clades provide an interesting system to test for patterns of phylosymbiosis. Within the teleost, some species have skin microbiomes that are species-specific and maintained across seasons [31], whereas in Atlantic cod (*Gadus morhua*), the sampling location affected microbiome structure [32]. Within Chondrichthyes (from here on elasmobranch fishes), the skin microbiome of the common thresher shark (*Alopias vulpinus*) was species-specific. There was higher microbiome similarity among individual thresher sharks, compared to individuals of another host and the surrounding seawater [6]. Similar to teleost, the sample location corresponded with the difference among skin microbiomes in blacktip reef sharks (*Carcharhinus melanopterus*) [33]. These results suggest that despite a lack of filtering features (i.e., mucus), elasmobranchs species select a specific microbiome, which may be linked to patterns of phylosymbiosis. In contrast, the gut microbiome of each of three different shark species was more similar to the gut microbiome of different teleost fishes than they were to each other [34]. This relationship suggests a dietary influence in the gut microbiome rather than phylosymbiosis. Whether the skin surface of the elasmobranchs compared with the teleost fishes influences the structuring of the microbiomes remains an outstanding question.

The skin of marine organisms is a dynamic interface with constant exposure to the surrounding environment, and therefore, predicting processes that govern microbiome assemblage in this space is complex. We developed a sampling framework to identify whether the skin microbiome from elasmobranch and teleost fishes exhibit phylosymbiosis, amidst the web of possible alternative drivers of microbiome structure, in both the taxonomic and gene function dimensions. In the marine environment, processes that influence functional gene composition in the microbiomes vary from processes that influence microbial taxonomic composition [35, 36]. Within an algae host microbiome, the functional genes, not the taxonomic composition, were species-specific [37]. Therefore, gene function may reveal processes underpinning the relationship between host microbiomes and should be considered when testing for phylosymbiosis. Consequently, we used shotgun metagenomics to explore whether patterns predicted by phylosymbiosis are apparent in the composition of potential gene functions, which, to our knowledge, remains untested.

Elasmobranchs investigated include the common thresher shark (*Alopias vulpinus*), whale shark (*Rhincodon typus*), leopard shark (*Triakis semifasciata*), and round ray (*Urolophus halleri*). Teleost fishes included the bay blennies (*Hypsoblennius gentilis*), California flounder (*Paralichthys californicus*), California killifish (*Fundulus parvipinnis*), shiner perch (*Cymatogaster aggregata*), and bay pipefish (*Syngnathus leptorhynchus*). First, we asked whether the skin microbiome of these marine fishes is host-specific. A central tenet of phylosymbiosis is that the variability of within-species microbiome similarity will be lower than that across host species; thus, we expect little variation among samples from the same species relative to samples among species. We extend the central tenet of phylosymbiosis to the elasmobranch and teleost clades. We determine whether the host evolutionary divergence extended to the accumulation of microbiome differences or if processes that result in patterns of phylosymbiosis appear to erode. Our results, from 38 total individuals from within 9 species, demonstrate that patterns of phylosymbiosis vary across clades and that convergent evolution of host traits may influence host microbiomes in teleost fish.

## Results

**Fig. 1:**
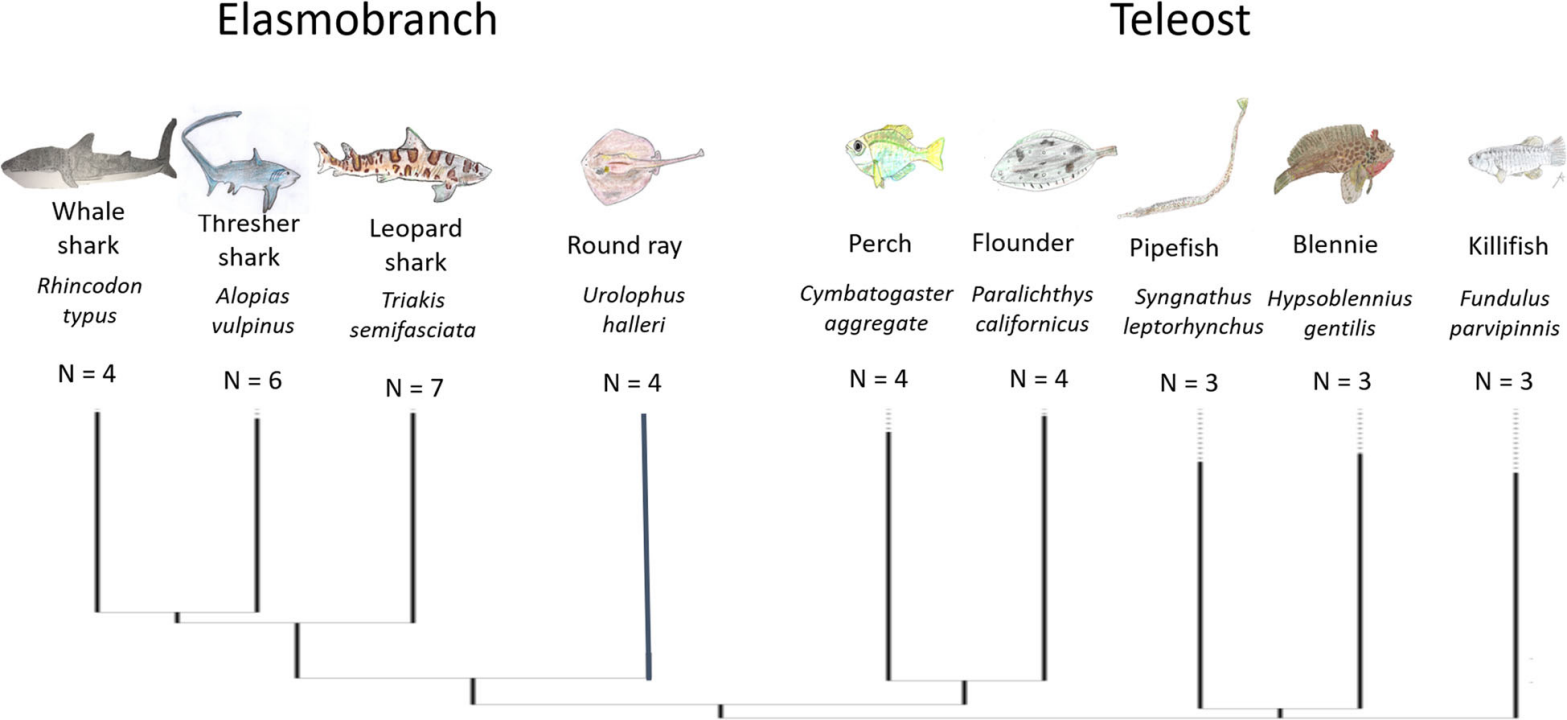
Hierarchical sampling design of comparisons among divergent vertebrate fish clades which include: four elasmobranch species (Chondrichthyes fishes) and five teleost species (Osteichthyes). *N* corresponds with the number of individual samples for each species. A total of 38 individuals were used in this analysis. Tree was built by sequence alignment of the COX1 gene of each species

Here, we present the distribution of microbiome diversity from the skin of two divergent fish clades (Fig. 1): the Chondrichthyes (cartilaginous) and Osteichthyes (bony) fishes which diverged ~ 420 mya. Our hierarchical sampling design included 3 shark and 1 ray species (*n* = 21) nested within the group elasmobranch and 5 bony fish species (*n* = 18; Table 1, Fig. 1) nested within in teleost group. Metagenomic libraries ranged in size from 58,623 to 3,482,509 reads (Table 1).

**Table 1:**
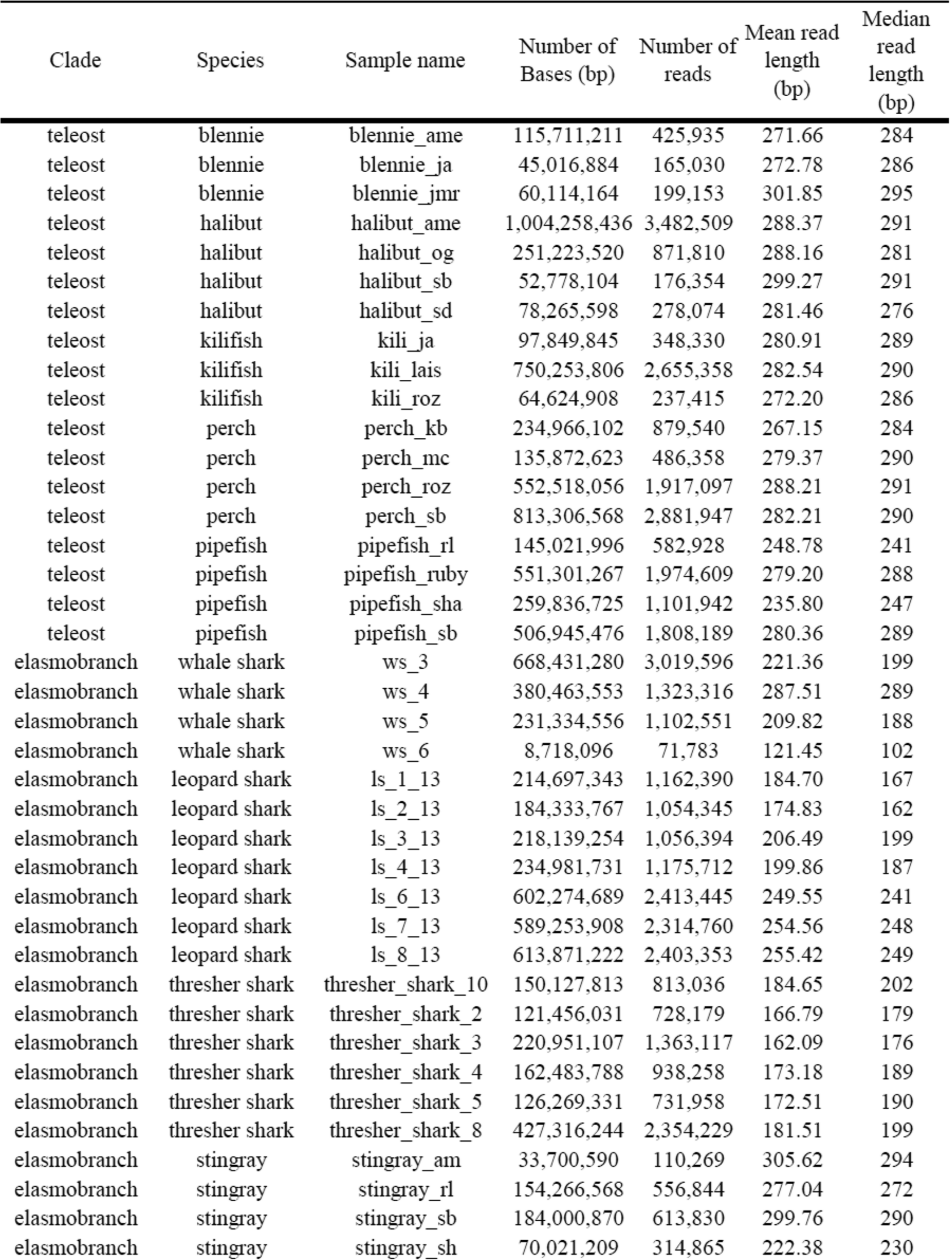
Metagenomic samples and sequence information. Sequence information is post quality control data

### Composition of the skin microbiomes

**Fig. 2:**
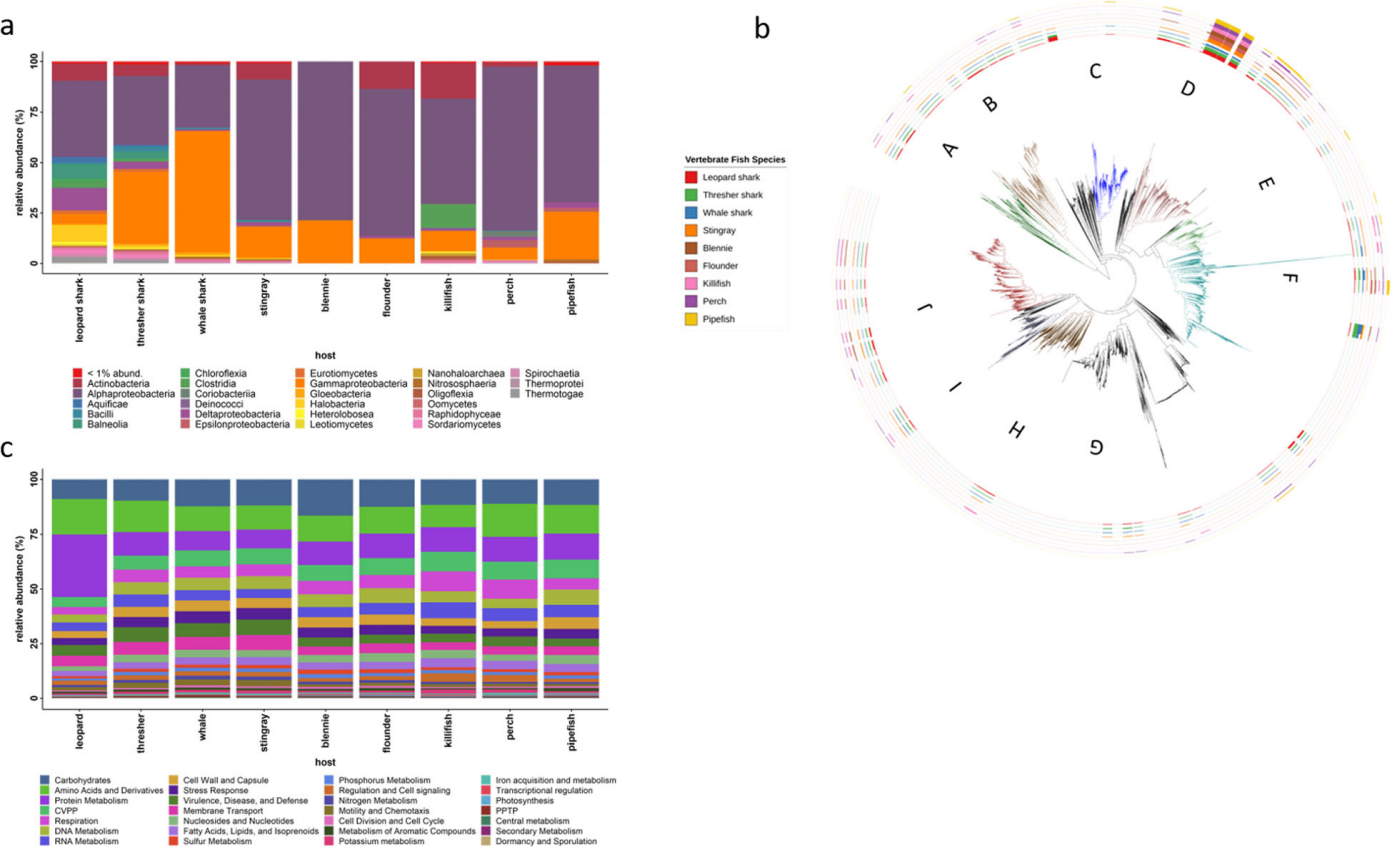
Taxonomic composition and phylogenetic placement of reads from metagenomics sequences from vertebrate fish skin microbiomes and level 1 gene function subsystems. **a**) The relative abundance of microbial classes identified from the metagenomic libraries of elasmobranch and teleost fishes. Taxonomic identity was assigned by aligning reads to conserved protein-coding genes [38] and mapping their placement onto a tree generated from the conserved reads. **b**) Phylogenetic diversity of elasmobranch and teleost skin microbiomes samples. Reference tree from PhyloSift which contains 4165 identified microbial species based on 37 conserved gene regions. Phylogenetic placement for conserved genes identified in elasmobranch or teleost fish microbiomes is labeled as bars on the periphery of the tree. Bar height represents the relative proportion of genes identified to that microbial leaf on the reference tree. Each circle represents an elasmobranch or teleost fish species. Letters identify the region of the tree where major microbial clades occur. Major clades include (A) Eukaryota superkingdom, (B) Archaea superkingdom, (C) Bacteroidetes, (D) Alphaproteobacteria, (E) Betaproteobacteria, (F) Gammaproteobacteria, (G) Bacillus, (H) Firmicutes, (I) Cyanobacteria, and (J) Actinobacteria. **c** The relative abundance of microbial gene function subsystems to the level 1 categorization identified from the metagenomic libraries in elasmobranch and teleost fishes

Using a reference tree of 37 conserved protein coding sequences from across the tree of life [38], we mapped the DNA reads from the metagenomes to compare microbial species represented in the elasmobranch and teleost fish microbiomes. The skin microbiomes of elasmobranch and teleost fishes have distinct taxonomic distribution patterns (Fig. 2a, b). Members of the skin microbiome span the breadth of the tree of life to include Eukaryota (0–7% total library abundance), Archaea (0–1.5% total library abundance), and Bacteria (92.6–100% total library abundance; Fig. 2b). The most abundant bacterial classes are Alphaproteobacteria, Gammaproteobacteria, and Actinobacteria; however, there were major differences across host clades. Within the elasmobranchs, there was the enrichment of Gammaproteobacteria from skin microbiomes of thresher and whale sharks, accounting for 35.7% and 60.3%, respectively, while Alphaproteobacteria accounted for 34.0% of the thresher microbiome and 30.8% of the whale shark microbiome. Leopard sharks had a more diverse distribution of sequences in each class with Alphaproteobacteria comprising 37.5% and Gammaproteobacteria comprising 5.0% of the microbiome. Other major groups contributing to the leopard shark microbiome included Deltaproteobacteria (10.8%), Actinobacteria (8.6%), and Halobacteria (8.2%). These classes were found in the thresher and whale shark microbiomes, but at lower proportional abundance. The stingray microbiome was more similar to several of the teleost microbiomes, being dominated by Alphaproteobacteria (69.5%) with Gammaproteobacteria only accounting for 15.3%. Teleost fish skin microbiomes were dominated by Alphaproteobacteria, ranging from 52.2% in killifish to 81.0% in perch. Gammaproteobacteria ranged from 5.9% in perch to 23.8% in pipefish. Actinobacteria comprise 13.6% of the flounder, 17.6% of the killifish, and 2.1% of the perch microbiomes, but was undetectable in the blennie and pipefish microbiomes. In addition to the Bacterial groups, we were able to identify various Eukaryota and Archaea in the skin microbiomes of elasmobranchs and teleost fishes, though these represented much less abundance relative to Bacteria (Fig. 2b). The elasmobranch microbiomes all harbored low relative proportions of archaeal groups, including Nitrososphaeria ranging from 1.0% in whale sharks to as low as 0.34% in leopard sharks, Thermoprotei which had an abundance of 0.5% in thresher sharks to 0.11% in stingrays, Thermoplasmata with 0.02% in stingrays to 0.04% in leopard sharks, and Nanohaloarchaea with 0.08% in thresher sharks to 0.02 in stingrays. In teleost fishes, only two archaeal groups were identified on two fish species, including Nanohaloarchaea in killifish (1.5%) and Nitrososphaeria in the pipefish (1.3%). Eukaryota was also found in the microbiomes of both elasmobranch and teleost fishes. Fungi groups dominated the Eukaryota group in elasmobranchs with Sordariomycetes having 2.3% total abundance in the leopard shark and 1.7% in the thresher shark, but only 0.3% and 0.04% in the whale shark and stingray, respectively. Eurotiomycetes was also in relatively high abundance on leopard sharks (1.7%) and thresher sharks (1.3%). An algal group, Raphidophyceae, was also found with a higher proportional abundance in thresher sharks (1.2%) and whale sharks (1.1%). The only Eukaryotic groups found in teleost skin microbiomes included Oomycetes (0.73%) and Raphidophyceae (1.5%) in killifish and Sordariomycetes (0.82%) and Raphidophyceae (0.27%) in perch microbiome. Blennie, flounder, and pipefish have no sequences matching Eukaryotic species present in the skin microbiome.

The functional potential of the skin microbiome of elasmobranchs and teleost fishes also varied (Fig. 2c). The most abundant group of gene functions (level 1 SEED subsystem) was protein metabolism with leopard sharks having 28.6% of the gene functions, while stingrays exhibited 8.5%. The most abundant gene functions for the other shark species included carbohydrate functions with 12.3% total gene functions for whale sharks and 11.9% for stringrays while amino acid-based gene functions were most abundant in thresher shark microbiomes (14.4%). In teleost, the largest average gene function abundance was carbohydrates with this function having the greatest relative abundance in blennie microbiomes (16.6%), flounder (12.5%), and killifish (11.7%). The most abundant gene functions for perch and pipefish were amino acid-based functions (15.0% and 12.9%, respectively).

### Host specificity of the microbiome

**Fig. 3:**
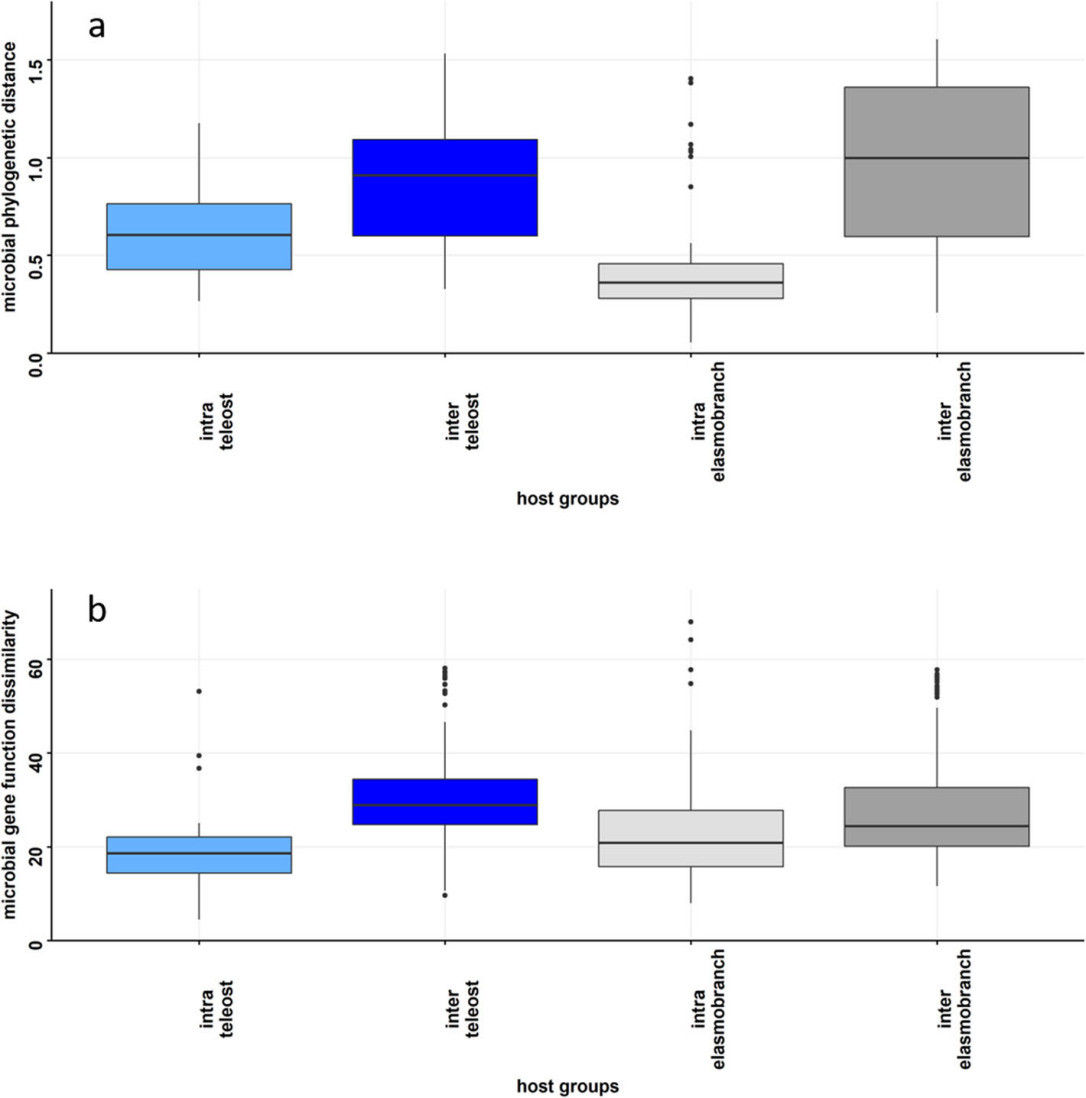
Box plots comparing the distribution of **a**) taxonomic beta diversity and **b**) gene function beta diversity within species and among species within clade. Intra-teleost = “within species of the teleost clade comparison”; inter-teleost = “among species within teleost comparison”; intra-elasmobranch = “within species of the elasmobranch clade comparison”; inter-elasmobranch = “among species of elasmobranch comparison.” Box plots represent the median with the 2nd and 3rd quantile represented within the box while whiskers represent the 1st and 4th quantile measures. All points beyond whiskers represent outlier samples. Statistical evaluation was only performed on within clade comparisons (ie. intra-elasmobranch:inter-elasmobranch). All statistical comparisons were significant

We hypothesized that the taxonomic and functional compositions of the microbiomes would be host-specific, i.e., microbiomes sampled from individuals of the same host species will be more similar than microbiomes sampled from individuals of a difference host species. To address this question, we first compared the similarity of microbiome composition within and across host species within their respective clades (i.e., within and among teleost species). Taxonomic similarity within host species was higher than compared with the microbiomes among host species (Kruskal-Wallis test – teleost: *χ*^2^_df = 1_ = 14.01, *p* < 0.001; elasmobranchs *χ*^2^_df = 1_ = 40.53, *p* = 0.01; Fig. 3a). Functional gene similarity followed a similar pattern with samples from within host species having greater similarity than samples among host species within their respective clade (teleost: *χ*^2^_df = 1_ = 27.17, *p* < 0.001; elasmobranch: *χ*^2^_df = 1_ = 11.93, *p* = 0.005).

### Clade specificity of the microbiome

**Fig. 4:**
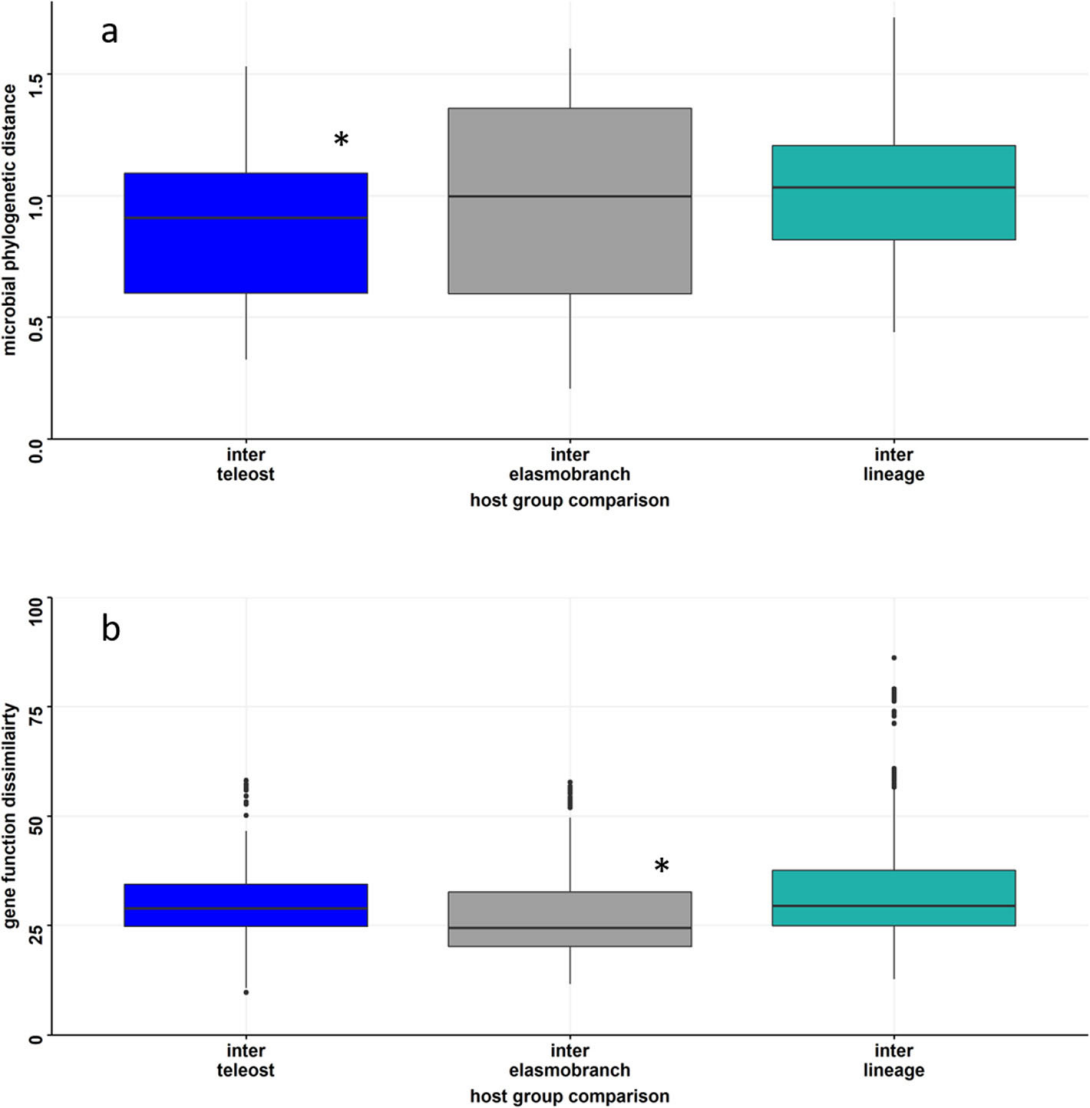
Distributional comparison of beta diversity for inter-clade (elasmobranch species and teleost comparisons) and intra-clade comparisons for both teleost and elasmobranch species (i.e., leopard shark–whale shark comparisons) for **a**) the taxonomic composition and **b**) gene function composition. Blue corresponds to teleost, grey to elasmobranch, and teal to among clade comparison (i.e., leopard shark–pipefish comparison). Box plots represent the median with the 2nd and 3rd quantile represented within the box with whiskers representing the 1st and 4th quantile measures. All points beyond whiskers represent outlier samples. Asterisk (*) denotes a significant difference (*p* < 0.05) relative to the inter-lineage comparison

We extended the microbiome analysis to the clade partition to account for the increased evolutionary history. If patterns of microbiome similarity are the result of processes consistent with phylosymbiosis theory, the average species pairwise similarity within each clade (intra-clade) is predicted to be greater than pairwise species comparisons among clades (inter-clade). The phylogenetic similarity of the microbiome shows that intra-elasmobranch similarity is not different than inter-clade comparisons; however, teleost fishes (mean phylogenetic distance = 0.79 ± S.E. 0.1: Fig. 4a; phylogenetic) have significantly lower microbiome phylogenetic distances relative to the inter-clade comparison (mean phylogenetic distance = 1.0 ± 1.6; Tukey’s post-hoc_intra-fish:interclade_, *p* = 0.03). For functional gene comparisons between clades, we find no difference in mean similarity scores between intra-clade and inter-clade pairwise comparisons (Fig. 4b).

**Fig. 5:**
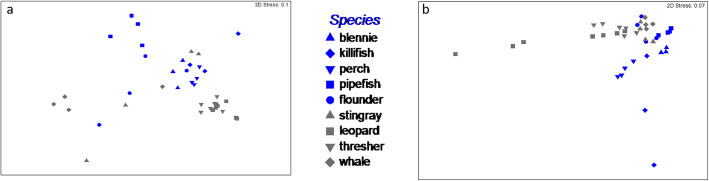
MDS ordination of the microbial community for **a**) taxonomic composition based on KR distance and **b**) gene function composition based on Bray-Curtis similarity. Grey corresponds to elasmobranch species and blue corresponds to teleost fish species

We performed an additional analysis to account for the high intra-species variability using a null-model approach on permuted distance matrices. The phylogenetic composition of the microbiome formed clade groups in the ordination space (Fig. 5a). However, several species within each clade had microbiomes that were more similar to a species across clades; thus, there was a lack of a clade signal (Table 2; phylogenetic, species effect: pseudo-F _df = 7,30_*=* 6.44, *p* < 0.01). For instance, the killifish microbiome was phylogenetically more similar to the thresher shark than to the founder (phylogenetic distance of killifish-flounder = 0.98 KR distance; killifish-thresher = 0.92 KR). Blennie microbiomes were more similar to leopard shark and stingray microbiomes than to pipefish (blennie-stingray = 0.92 KR; blennie-pipefish = 0.97; Supplemental Table1).

**Table 2:**
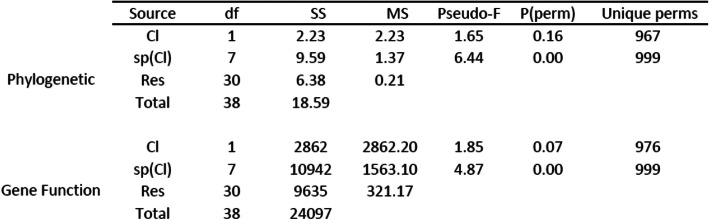
PERMANOVA output for taxonomic and gene function community dimension comparisons. Cl: clade; Sp: species

*Cl* clade, *Sp* species

A similar analysis was conducted on the functional gene composition, which showed microbiomes formed host-specific groups (pseudo-F _df = 7,30_*=* 4.87, *p* < 0.01). However, the elasmobranch and teleost clades were not significantly different (Fig. 5b; Table 2). The lack of clade effect is due to some host species within a clade having a functional gene composition of the microbiome that is more similar to a microbiome of a host species in the other clade. The functional gene composition of flounder microbiomes was more similar to that of thresher shark (25.01) and whale shark (18.6) compared with other teleost microbiome, such as killifish (40.1) or perch (30.0), estimated using Bray-Curtis dissimilarity (Supplemental Table 1). The lack of observed clade effect is contrary to the prediction of phylosymbiosis. Geographic distance could be proposed as a reason for the similarities across clades being identified. However, this was not the case, as the teleost fish and stingray were collected at the same location and the leopard sharks, thresher sharks, and whale sharks were geographically more distant. Thus, the whale shark and flounder were most geographically distinct, but show higher microbiome similarity than predicted, and the converse was identified for the teleost fish (i.e., distinct microbiomes, while they were collected at the same location).

### Microbiome and host phylogenetic distance

**Fig. 6:**
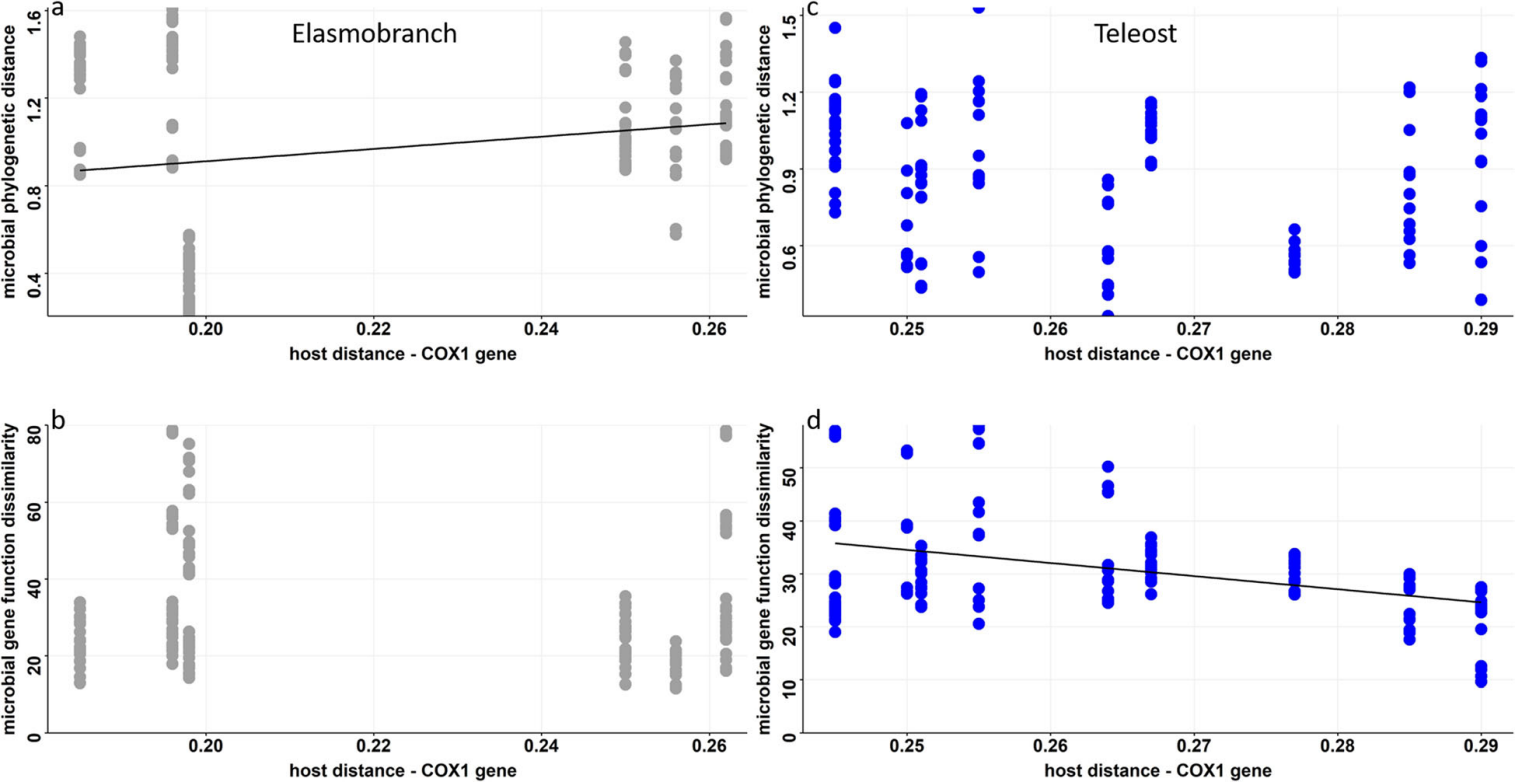
Comparison host genetic divergence to **a**) elasmobranch microbiome phylogenetic distance, **b**) elasmobranch gene function dissimilarity, **c**) teleost microbiome phylogenetic distance, and **d**) teleost gene function dissimilarity. The host genetic differences (*x*-axis) were calculated as the difference in the COX1 gene

Phylosymbiosis argues that increasing host evolutionary distance results in accumulated microbiome divergence (increasing dissimilarity or distance). To test this hypothesis, we compared the host’s evolutionary distance (based on the phylogenetic assessment of the COX1 gene) to the divergence of the microbiome for each clade (note that the clades were compared separately because of variations in the evolutionary clock). In elasmobranchs, we found a significant increase in microbiome distance with increasing host evolutionary distance (Fig. 6a; *F*_df = 1__,160_ = 7.09, Adj-R2 = 0.04, *p* < 0.01), supporting phylosymbiosis. For teleost, there was no significant relationship between host distance and microbial phylogenetic distance. In contrast, the gene function comparison showed the reverse trend. There was a lack of relationship between host evolutionary distances for gene function of the microbiomes for elasmobranchs (Fig. 6b), whereas there was a relationship between the gene function of the microbiomes and the evolutionary distance of the teleost fishes (*F*_df = 1__,127_ = 22.9, Adj Rsq = 0.15, *p* < 0.01). The decreasing slope indicates that functional similarity is increasing with increasing host evolutionary distance.

## Discussion

Elasmobranch fishes, of the Chondrichthyes clade and teleost fishes of the Osteichthyes clade, diverged approximately 420 mya [26], resulting in morphological and physiological differences, and here, we show this divergence extends to the relationship of the host with its skin microbiome. We found host species to harbor unique microbial symbiotic communities, both taxonomically and at the functional gene level. Host-specific microbiomes are a common pattern in nature, occurring in many host organisms, including coral reef fishes, nasonia wasps, mosquitos, mice, and drosophila [10, 24]. Host specificity of the functional gene composition of the microbiome communities is described for a few marine organisms, including the skin of the common thresher shark [33], and an algal species, *Ulva australis* [37]. Here, we show that the microbial functional genes are specific to a further eight marine host species.

Microbiome community similarity is predicted to decrease with the increasing evolutionary divergence of host organisms [10]. Therefore, we predicted that host species microbiomes would be more similar among clades than across clades. We found that elasmobranchs species’ microbiomes did not vary from inter-clade (elasmobranch to teleost microbiomes comparison), but the teleost fish microbiomes did exhibit a lower microbiome phylogenetic distance relative to the inter-clade comparison. However, some species within each clade had microbiomes that were more similar to species across clade boundaries, thus a non-significant comparison. For functional genes, elasmobranchs exhibited a lower microbiome functional dissimilarity within clades compared with between clades, but this was not the case for teleost fish microbiomes. Most phylosymbiosis studies have not compared across clades [10, 17], making this, to the best of our knowledge, the first study to do so.

We next tested for the effects of host evolution within clade on the microbiome, predicting that species with a more recent common ancestor would have a microbiome that is more similar in composition. With the methods used, we observed that elasmobranch fishes exhibited increased microbiome divergence with increasing host difference. Teleost fishes, however, exhibited no relationship in microbiome divergence and host difference. In fact, for teleost, the slope trended in the opposite direction relative to predictions (albeit a non-significant slope was reported). The phylogenetic assessment of the elasmobranch species based on the COX1 gene suggests they have a more recent common ancestor relative to the teleost fish species. However, we note caution in the interpretation of the results based on the COX1 gene, as the mitochondrial DNA among Chondrichthyes fish species accumulates nucleotide differences at rates much slower than their Osteichthyes fish counterparts [39]. Therefore, we suggest that the elasmobranch species emerged earlier than the teleost species and that the microbial skin species and elasmobranchs have evolved in a manner consistent with phylosymbiosis.

Teleost fish, however, lack a consistent phylosymbiosis relationship, which may be the result of convergent evolution of traits in skin features selecting for specific microbiome inhabitants. The increasing microbiome similarity (for both phylogeny and functional genes of the microbiome) with increasing host distance suggests convergent evolution for traits that fish use to select and maintain a microbiome [40, 41]. Similarly, Chiarello et al. [24] found weak support for phylosymbiosis in coral reef fishes but did not analyze the microbial functions. Teleost fishes are covered in mucus of varying chemical compounds and thickness [28, 42], which influences microbiome composition depending on the presence of host immunological factors and mucus chain sugar residues [43]. In addition, the epidermal mucus from teleost harbor anti-microbial properties [44, 45]. Thus, the microbiome requires similar functional genes to utilize the mucus and evade the anti-microbial properties, possibly leading to the convergent evolution of the microbiome on teleost fish.

The lack of a pattern for phylosymbiosis in the genes required to live on elasmobranch fishes may occur because mucus is not a selective mechanism. There are low amounts of mucus excreted onto the skin surface [29], except for stingrays [46]. The microbes on the elasmobranchs are not utilizing mucus but using the skin surface as a habitat. In this case, the microbes require unique traits to attach and establish a biofilm on each of the elasmobranchs. In support, we found gene functions, which could determine different lifestyles of the microbes, to vary in relative abundance across the two fish groups. For instance, the relative proportions of sequences within the functional pathways, motility and chemotaxis, and membrane transport was higher for elasmobranchs compared with teleost fishes. These are genes that would be used by microbes to move and uptake nutrients, whereas teleost fishes had a higher proportional abundance of sequences within the protein metabolism (when leopard sharks are excluded—28.6% of total abundance) compared to elasmobranch fishes, and potentially these genes are used for breaking down the mucus component excreted by the teleost fish.

Microbiome patterns observed in this study could also be the result of conserved symbiosis from a distant shared ancestor of elasmobranch and teleost fishes, whereby some interactions have been maintained and others have been lost. Such processes have been hypothesized for the convergence observed in some teleost fish and mammal gut microbiome [47], in which teleost fishes formed distinct symbiotic relationships, that remained conserved as mammals radiated from the bony fish clade. Similarly, human and old-world monkey gut microbiomes did not show phylosymbiosis as a result of host adaption for an omnivorous diet [48]. The adaptive process is hypothesized to be the result of aquiring symbionts, which evolved before the evolution of the host organism. Groussin et al. [13] showed that host organisms which share a common ancestor more recently have stronger patterns of phylosymbiosis in the gut microbiome while increasing time since shared ancestry corresponded with a decrease in phylosymbiosis. They attribute this relationship to dietary switching, which has led to acquiring microbial symbionts that evolved independently of the host organism; therefore, host species with common ancestors that share dietary constraints have more similar gut microbial communities, similar to our observation of the lack of relationship between the teleosts and microbiome. In addition, host diet was a better predictor of microbiome composition than was phylogenetic placement (Muegge et al. 2011). By mapping conserved gene sequences on the tree of life, we observed conserved and specific microbial classes across the fish clades. The divergent microbial species suggest a possible co-evolutionary interaction between microbial species and the elasmobranch host.

The lack of consistency in the relationship of microbiomes across clades could also be the result of eco-environmental effects, such as biogeography. Capture sites of blacktip reefs sharks (*Carcharhinus melanopterus*) accounted for high variation in microbiome composition [33]. However, the observed patterns are not consistent with the location of sampling in our study. For instance, the stingrays were collected in the San Diego region, as were the leopard sharks; however, the stingray microbiomes were more similar to the whale shark microbiomes, which were collected in La Paz, Mexico (Supplemental Table 1). Similarly, environment has been shown to be linked with the skin microbiomes of teleost fishes [49, 50] and elasmobranchs [33], but these studies have focused on populations of a single species or biogeography, thus limiting insight into possible phylogenetic structure. Our study has leveraged several species, which exhibit varying geography, environment, and trophic positioning. If the environment is a stronger driver of microbiome composition than phylogeny, we would expect all teleost fish and the stingray to have a similar microbiome, as all samples were collected in San Diego, USA. However, this was not observed. The trophic position of the host influences the microbiome structure as well [47]; however, we observed that whale sharks, which are filter feeders (omnivorous), and stingrays, which are benthic carnivores, had similar microbiomes. Whereas stingrays and leopard sharks both consume benthic invertebrates, but their microbiomes were dissimilar. The similarity of thresher shark and killifish microbiomes further contradicts the trophic hypothesis. Therefore, our study suggests that the taxonomy of the elasmobranch microbiome follows the phylosymbiosis model, while the teleost microbiomes appear to be converging.

## Conclusion

While host-specific patterns of microbiome assemblage are commonly observed in nature, processes which govern these microbiome assemblage patterns remain poorly understood and debated [10, 15]. Here, we extend insight into host microbiome assemblage by examining patterns on the skin surface of teleost and elasmobranch fishes, a split which represents arguably the most important diversification event among vertebrates. In addition, we extended this analysis to examine patterns of functional gene composition in the skin surface microbiome. While phylosymbiosis represents an obvious null model from which to evaluate host microbiome assemblage, the lack of a consistent pattern combined with the emergence of trends in functional gene composition suggest processes underlying assemblage patterns are operating on scales which are not well understood. For instance, while the taxonomic composition of teleost fishes lack a pattern consistent with phylosymbiosis, the function gene composition reveals the possibility for convergence, a pattern evidenced by the increased functional gene similarity in the microbiomes with increasing host distance. In contrast, elasmobranchs have a pattern of the taxonomic composition of the microbiome consistent with phylosymbiosis yet lack a pattern of phylosymbiosis in the functional gene composition. The discrepancies in skin microbiome pattern across these two fish clades is the result of processes acting on the microbiomes which operate at varying scales, a point that to date has rarely been considered in the theory of microbiome research.

## Methods

### Sample collection

Common thresher shark (Table 1; *n* = 6) collection and processing are described in detail in Doane et al. [6]. Briefly, samples were collected in collaboration with NOAA Southwest Fisheries’ annual thresher shark survey in September 2013. The leopard shark (*n* = 7) samples were collected from La Jolla, CA, USA, in September 2013 using a hook and line methods, with the shark brought into the boat for microbial sampling. Whale shark (*n* = 4) microbial samples were collected from La Paz, Mexico, in February 2014. The skin microbiota was obtained via a two-way modified syringe in which expelled water is recollected in the backside of the syringe [6]. Thresher and leopard shark samples were collected while the animal was in the boat; however, whale shark samples were collected while freediving alongside the animal. This method is possible for sampling surfaces in the water due to the enclosed compartment created when the syringe is pressed firmly against the skin surface of the shark, keeping seawater outside the sampled area. The round ray and all teleost fish samples were collected using a beach seine over a seagrass bed in Mission Bay, San Diego, USA, in February 2017. The round rays (*n* = 4) were put into 5-L shallow bins filled with bay seawater until ready to sample. Rays were lifted just out of the water, and the 2-way syringe was used to collect microbes from the dorsal surface just posterior of the eyes. The 2-way syringe was preloaded with a sterile PBST solution [51]. The other four teleost fish species were sorted into buckets of bay seawater and identified. Single individuals of all species (except bay pipefish, in which four individuals were placed into bottles together) were sorted into their own 500 mL bottles containing PBST and shaken lightly. The bottle was emptied (fish included) through a net to catch the fish for release, while the solution was caught by a clean 500-mL tri-pour. Our sample size was similar to other microbial analyses of marine fishes [24]. The water from the sharks or the solution from the ray and teleost fishes were passed through a 0.2-μm Sterivex filter (Millipore) to capture microbial cells. Filters from all specimens were stored dry at − 20 °C until extraction. All samples were collected from the dorsal skin surface along the base of the first dorsal fin when possible. Stingray samples were collected from the center of the dorsal surface. Teleost samples were collected from the entire outer surface as individuals were submerged in sample solution because they were too small to use the super-sucker technique.

### DNA extraction and metagenomic sequencing

DNA was extracted from the microbes captured on the filter using a modified column purification protocol from Macherey-Nagel Nucleospin Tissue kit as described in Doane et al. [6]. In brief, 720 μL of T1 buffer and 90 μL of Proteinase K (2.5 mg/mL) were added to all Sterivex filter cartridges. The ends were then sealed and set to incubate overnight at 55 °C with rotation. DNA extraction followed the Nucleospin Tissue protocol. All DNA samples (except the common thresher shark which is described in [6]) were prepared for sequencing using the Accel-NGS 2S Plus DNA kit (Swift Biosciences, Ann Arbor, MI, USA) for paired-end sequencing with the Illumina MiSeq v3 600 cycle (San Diego, CA, USA). The sequencing of the teleost fish and stingray samples was conducted by students in the San Diego State University Ecological Metagenomic course [52]. The thresher, whale, and leopard shark samples were sequenced in several Illumina runs with samples mixed with microbiomes from a range of projects, e.g., water column and kelp forests.

### Library quality control and annotation

All libraries (including common thresher shark), were cleaned using PRINSEQ software [53] to filter out all artificial duplicate reads, sequences less than 70 base-pairs, sequences with base quality averaging a score of less than 25, and any sequence with more than a single N (ambiguous base). Sequences were paired using software PEAR, a paired-end read merger [54]. Sequences in final libraries contain all paired sequences, all singleton sequences passing quality control in PRINSEQ, and all forward unpaired reads identified with PEAR.

A total of 38 metagenomic libraries were used in the analysis. Function genes and phylogenetic microbiome diversity were annotated in the following manner. Functional genes were assigned using SUPERFOCUS [55], which first identifies the taxonomic assignment of the sequence using k-mer profiling (annotated June 2017), then builds a database of only those identified taxa’s genomes to align and assign potential gene function to each read using RAPSearch alignment [56]. The functional assignments are described in a hierarchical manner [57]. We collapsed all data into the Level 3 subsystems (e.g., protein secretion systems, type VII), which describes the specific category of potential encoded protein of the gene (from here on referred to as gene function). SUPERFOCUS was a highly ranked tool for accurately annotating metagenomes by the 2017 Critical Assessment of Metagenomic Interpretation (CAMI) group [58]. Phylogenetic placement of the microbiome samples was conducted using marker genes through the PhyloSift framework [38]. In brief, the PhyloSift software finds marker genes using homology-based searching within metagenomes. It works in three steps: homology-based matching of metagenomics reads to reference database using LAST, reference multiple alignments with HMMER 3.0, and placement into a phylogenetic reference tree using pplacer (annotation March 2018). The resulting output is a JPLACE file format [59] for each sample, and manipulation of this file format was conducted using the guppy software (http://erick.matsen.org/pplacer/generated_rst/guppy.html). Tree visualization was conducted using iTOL v3 [60]. Query read placement and abundance were visualized on the periphery of the tree to visualize their relative abundance and position among the reference tree. Abundance matrices were derived from the number of identified sequences that most closely aligned and taxonomically identify assigned to the taxa of the closest aligned reference sequence. Each of the microbial community dimensions (gene function, and phylogeny) were organized into a data matrix. The phylogenetic tree was converted to data matrices with samples as rows and columns containing sequence identification, taxonomic assignment of leaf, pendant length, distal length, and maximum likelihood score [59]

### Statistics

Gene function matrices were standardized to the summed total of each sample. The phylogenetic matrix was not standardized, as PhyloSift provides a weighted normalization output. The similarity in microbiomes was calculated for gene functions using Bray-Curtis dissimilarity matrices and for phylogenetic distance using Kantorovich-Rubinstein metric (KR-distances from here on) [61]. For aims one and two, we determined whether host species microbiomes were distinct and whether phylosymbiosis signals were apparent in the elasmobranch-teleost fish comparison. β-diversity was defined as Bray-Curtis dissimilarity (gene function) and KR distance for phylogenetic composition. Kruskal-Wallis (kruskal.test; R) was used to test for differences within and among species and within and among clades. In addition, we tested for a difference between the mean distance among species within clade and the mean distance among species across clade. A Dunn test (dunn.test; R) was performed as a post-hoc test to identify pair-wise differences among clades using a Bonferroni *p* value correction. We tested for differences in β-diversity among clade (elasmobranch versus teleost fishes) and species (i.e., thresher, whale, or pipefish) using a two-factor nested PERMANOVA. A permutational *t* test was used to determine which species were causing the differences when the main effect test was significant. Nonmetric multidimensional scaling (nMDS) was used to visualize the microbiome (gene function and phylogenetic) dissimilarity or distance in ordination space. To test if skin microbiome composition was linked with host phylogeny, we calculated host distance by aligning the cytochrome c oxidase I (COX1) gene of each species using Clustal Omega [62] on the EMBL-EBI server. Default parameters were used. COX1 genes were downloaded from NCBI. The COX1 gene has been used because it represents the only host gene publicly available for host phylogenetic comparison. We determined the relationship of host distance to microbiome similarity using linear modeling (lm; R).

All statistics were run using R (v3.5.1) and Primer package 6 (v6.1.15) with PERMANOVA+ (v1.0.5). All figures were generated ggplot2 package in R (v3.5.1).

## Supplementary information

**Additional file 1 Supplemental Table 1.** All pairwise comparisons among species across all community dimensions. P (perm) is the calculated p-value based on permuted values. BC similarity is Bray-Curtis similarity. Phylogenetic distance is the KR distance method.

## Data Availability

All sample are available through the MG-RAST server (https://www.mg-rast.org/).
