# Supplementary material for: The skin microbiome of elasmobranchs follows phylosymbiosis, but in teleost fishes, the microbiomes converge
Source: Microbiome. 2020 Jun 13;8:93. doi: 10.1186/s40168-020-00840-x (PMC7293782; doi:10.1186/s40168-020-00840-x)
Supplement: Supplementary file 2 — Additional file 1 Supplemental Table 1. All pairwise comparisons among species across all community dimensions. P (perm) is the calculated p-value based on permuted values. BC similarity is Bray-Curtis similarity. Phylogenetic distance is the KR distance method. [file 40168_2020_840_MOESM1_ESM.pdf]

		Phylogenetic		Gene Function	
Group	Species	P(perm)	Distance	P(perm)	BC
shark	ls, stingray	**0.00**	1.15	**0.02**	41.48
	ls, ts	**0.02**	0.35	**0.01**	34.59
	ls, ws	**0.01**	1.34	**0.02**	42.03
	stingray, ts	**0.01**	1.08	**0.01**	24.52
	stingray, ws	0.16	1.06	0.33	18.18
	ts, ws	**0.01**	1.25	**0.01**	23.86
inter	blennie, ls	**0.01**	0.92	**0.02**	45.23
	blennie, stingray	0.25	0.92	**0.04**	30.25
	blennie, ts	**0.02**	0.86	**0.01**	22.93
	blennie, ws	0.06	1.13	**0.04**	26.01
	flounder, ls	**0.00**	1.12	**0.02**	41.78
	flounder, stingray	0.82	0.92	0.79	19.61
	flounder, ts	**0.01**	1.04	**0.01**	25.07
	flounder, ws	0.14	0.99	0.45	18.55
	ls	**0.01**	0.96	**0.02**	54.71
	kilifish, stingray	0.17	1.03	**0.04**	39.61
	ts	**0.01**	0.92	**0.01**	43.14
	kilifish, ws	0.06	1.33	**0.03**	41.76
	ls, perch	**0.00**	0.74	**0.02**	41.13
	ls, pipefish	**0.00**	1.25	**0.01**	45.87
	perch, stingray	**0.03**	0.89	**0.02**	30.10
	perch, ts	**0.01**	0.71	**0.01**	29.12
	perch, ws	**0.04**	1.16	**0.03**	32.27
	pipefish, stingray	**0.02**	1.17	**0.02**	24.47

	pipefish, ts	**0.01**	1.17	**0.01**	30.16
	pipefish, ws	**0.03**	1.18	**0.03**	22.36
fish	blennie, flounder	0.32	0.82	0.05	24.36
	blennie, kilifish	0.20	0.70	0.09	39.60
	blennie, perch	**0.02**	0.56	**0.03**	30.25
	blennie, pipefish	**0.03**	0.97	**0.03**	22.93
	flounder, kilifish	0.10	0.98	**0.02**	40.11
	flounder, perch	**0.03**	0.84	**0.03**	30.03
	flounder, pipefish	**0.03**	0.98	**0.05**	21.65
	kilifish, perch	**0.03**	0.59	0.11	34.06
	kilifish, pipefish	**0.03**	1.14	**0.03**	41.47
	perch, pipefish	**0.03**	1.04	**0.04**	32.12
